# 5-ALA Photodynamic Therapy Induces Competing Death and Survival Pathways in Glioblastoma Cells

**DOI:** 10.3390/cimb48070689

**Published:** 2026-07-03

**Authors:** Julia Inglot, Dorota Bartusik-Aebisher, Joanna Katarzyna Strzelczyk, Angelika Myśliwiec, Klaudia Dynarowicz, Dorota Hudy, Oliwia Trzaskoś, Jacek Tabarkiewicz, Aleksandra Kawczyk-Krupka, Magdalena Moś, David Aebisher

**Affiliations:** 1 English Division Science Club, Faculty of Medicine, Collegium Medicum, University of Rzeszów, 35-310 Rzeszów, Poland; inglotjulia@gmail.com; 2 Department of Biochemistry and General Chemistry, Faculty of Medicine, University of Rzeszów, 35-310 Rzeszów, Poland; dbartusikaebisher@ur.edu.pl (D.B.-A.); amysliwiec@ur.edu.pl (A.M.); kdynarowicz@ur.edu.pl (K.D.); 3 Department of Medical and Molecular Biology, Faculty of Medical Sciences in Zabrze, Medical University of Silesia in Katowice, 19 Jordana St., 41-808 Zabrze, Poland; jstrzelczyk@sum.edu.pl (J.K.S.); dorota.hudy@sum.edu.pl (D.H.); 4 Department of Human Immunology, Institute of Medical Sciences, Medical College of Rzeszów University, University of Rzeszow, 35-959 Rzeszów, Poland; otrzaskos@ur.edu.pl (O.T.); jtabarkiewicz@ur.edu.pl (J.T.); 5 Department of Internal Diseases, Angiology and Physical Medicine, Center for Laser Diagnostics and Therapy, Faculty of Medical Sciences in Zabrze, Medical University of Silesia, 40-055 Katowice, Poland; akawczyk@gmail.com; 6 Department of Internal Diseases, Angiology and Physical Medicine, Center for Laser Diagnostics and Therapy, Medical University of Silesia, 41-902 Bytom, Poland; magdalenamos99@gmail.com; 7 Department of Photomedicine and Physical Chemistry, Faculty of Medicine, University of Rzeszów, 35-959 Rzeszów, Poland

**Keywords:** glioblastoma multiforme (GBM), photodynamic therapy (PDT), 5-aminolevulinic acid (5-ALA), apoptosis, ferroptosis

## Abstract

Glioblastoma multiforme (GBM), isocitrate dehydrogenase (IDH)-wildtype, is the most aggressive primary malignant tumor of the central nervous system, characterized by poor prognosis and high recurrence rates despite standard multimodal treatment. This study investigates the molecular response of glioblastoma cells to 5-aminolevulinic acid (5-ALA)-based photodynamic therapy (PDT), focusing on gene expression changes associated with apoptosis, ferroptosis, and oxidative stress. Human glioblastoma T98G cells were treated with 5-ALA followed by light irradiation, and gene expression was analyzed using RT-qPCR. PDT induced moderate upregulation of pro-apoptotic genes (*BAX*, *CASP3*, *FAS*) alongside increased expression of the anti-apoptotic gene *BCL2*, indicating simultaneous activation of cell death and survival pathways. Ferroptosis-related genes showed mixed responses, with slight upregulation of *ACSL4* and downregulation of *GPX4*, suggesting increased susceptibility to lipid peroxidation. The most significant change was observed in *GCH1* expression, reflecting activation of oxidative stress response mechanisms. However, none of the observed changes reached statistical significance, likely due to the limited sample size. These findings demonstrate that PDT induces a complex and dual biological response in glioblastoma cells, involving both cytotoxic and adaptive mechanisms. This may limit therapeutic efficacy and contribute to treatment resistance. The results support the rationale for combining PDT with targeted molecular therapies aimed at inhibiting antioxidant defenses and anti-apoptotic pathways. Additionally, personalized therapeutic strategies based on tumor molecular profiles may enhance treatment outcomes. Further studies with larger sample sizes and functional validation are required to confirm these preliminary observations.

## 1. Introduction

Glioblastoma multiforme (GBM), IDH-wildtype, is the most common and most aggressive primary malignant tumor of the central nervous system. Despite standard treatment involving maximally safe surgical resection, radiation therapy, and temozolomide chemotherapy, the prognosis for patients remains poor, with a median overall survival of approximately 14–18 months. The high recurrence rate, particularly within the postoperative bed and the tumor infiltration zone, indicates the limited efficacy of cytoreductive surgery alone and the need to seek local treatment methods targeting residual cells [1,2,3,4,5].

One promising adjuvant strategy for the treatment of glioblastoma is photodynamic therapy (PDT), which relies on the interaction of a photosensitizer, light of an appropriate wavelength, and molecular oxygen. Activation of the photosensitizer leads to the generation of reactive oxygen species, resulting in damage to cellular structures and the death of cancer cells while relatively sparing normal tissues. Of particular importance in neuro-oncology is 5-aminolevulinic acid (5-ALA), which is a precursor of protoporphyrin IX (PpIX). The selective accumulation of PpIX in glioma cells enables both intraoperative tumor visualization and the use of 5-ALA in therapeutic strategies, including PDT and interstitial PDT (iPDT) [6,7,8,9].

Current clinical trials and reviews indicate that 5-ALA-based PDT may be a safe and potentially effective adjunct to the treatment of malignant gliomas, particularly in the context of local control and the treatment of recurrent lesions. It has also been demonstrated that the combination of 5-ALA fluorescence-guided resection with PDT may be associated with improved survival without a significant increase in severe complications, although further randomized trials in larger patient cohorts are necessary [10,11,12].

Recent comprehensive reviews have further highlighted the growing interest in PDT as an adjunctive strategy for glioblastoma treatment. In a recent narrative review, Price et al. summarized the current state of clinical PDT applications in glioblastoma, emphasizing encouraging results in local tumor control and progression-free survival while also identifying major barriers to broader implementation, including limited light penetration, heterogeneous photosensitizer accumulation, tumor hypoxia, and the lack of standardized treatment protocols. The authors additionally stressed the need for larger prospective clinical studies to establish the optimal role of PDT within contemporary multimodal glioblastoma management [13].

Complementing these clinical perspectives, Jia et al. focused on nanotechnology-enhanced PDT approaches designed to address one of the greatest challenges in neuro-oncology—the blood–brain barrier (BBB). Their review highlighted the potential of nanoparticles, liposomes, polymeric carriers, and targeted delivery systems to improve photosensitizer transport across the BBB, enhance tumor-selective accumulation, prolong circulation time, and increase photodynamic efficacy while minimizing off-target toxicity. These developments may help overcome several biological limitations currently restricting the therapeutic effectiveness of conventional PDT [14].

Furthermore, a recent systematic review by Netto et al. evaluated emerging minimally invasive laser- and light-based therapies for glioblastoma, including laser interstitial thermal therapy, interstitial photodynamic therapy, and other image-guided phototherapeutic approaches. The authors concluded that these technologies offer promising opportunities for treating recurrent, deep-seated, or surgically challenging tumors while potentially reducing treatment-related morbidity. However, they also emphasized the need for further clinical validation and long-term outcome studies before widespread adoption can be recommended [15].

Collectively, these recent reviews demonstrate that the field of glioblastoma phototherapy is rapidly evolving beyond conventional fluorescence-guided surgery toward increasingly sophisticated therapeutic applications. Advances in photosensitizer delivery, nanomedicine, image-guided light administration, and combination strategies integrating PDT with immunotherapy, targeted therapies, or other minimally invasive interventions may further enhance treatment efficacy and expand the clinical utility of PDT in neuro-oncology. Such developments provide a strong rationale for continued investigation of the molecular mechanisms underlying PDT responses in glioblastoma cells and for identifying biomarkers associated with treatment sensitivity and resistance.

Given these properties, 5-ALA-mediated PDT has emerged as a promising strategy for glioblastoma treatment. However, the molecular mechanisms underlying the cellular response to PDT remain incompletely understood. Therefore, the present study aimed to evaluate gene expression changes associated with apoptosis, ferroptosis, and oxidative stress following 5-ALA-mediated PDT in T98G glioblastoma cells.

Despite growing interest in 5-ALA-mediated PDT for glioblastoma, the molecular mechanisms underlying the cellular response to treatment remain incompletely understood. In particular, the relative contribution of apoptotic, ferroptotic, and oxidative stress-related pathways to PDT-induced cytotoxicity is still being investigated. Therefore, the primary aim of this study was to evaluate the expression of selected genes associated with apoptosis (*BAX*, *BCL2*, *CASP3*, *FAS*), ferroptosis and redox regulation (*ACSL4*, *GPX4*, *SLC7A11*), and oxidative stress adaptation (*GCH1*) in T98G glioblastoma cells following 5-ALA-mediated photodynamic therapy. We hypothesized that PDT-induced oxidative stress would modulate the expression of genes involved in both cell death and adaptive survival pathways, reflecting the complex biological response of glioblastoma cells to photodynamic treatment.

Recent studies indicate that the efficacy of PDT in glioblastoma multiforme is not limited solely to direct cellular damage but also involves the activation of multiple regulated cell death pathways. PDT-induced oxidative stress leads to the accumulation of lipid peroxides and may initiate the process of ferroptosis, which is closely associated with redox imbalance. Simultaneously, classical apoptosis mechanisms are activated, including the release of cytochrome c and the activation of caspases. Under conditions of severe cellular stress, necroptosis may also be activated. The multifaceted action of PDT helps overcome tumor resistance mechanisms to treatment [16,17,18].

Photodynamic therapy also plays a significant role in modulating the tumor microenvironment and activating the immune response. Damage to cancer cells leads to the release of DAMP molecules, such as calreticulin and HMGB1, which initiate immunogenic cell death and promote the activation of dendritic cells and T lymphocytes. Furthermore, PDT influences the expression of immune checkpoint molecules, including PD-L1, and the signaling of cytokines such as IL-6 and TGF-β, which play a key role in immunosuppression in GBM. It has been suggested that combining PDT with immunotherapy may enhance treatment efficacy by overcoming local immune tolerance [19,20].

**Figure 1:**
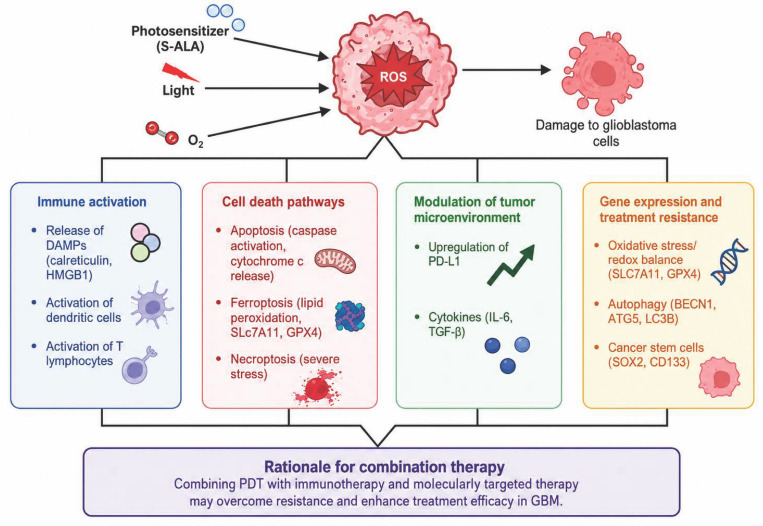
A simplified diagram of the mechanisms of PDT in glioblastoma. PDT, involving a photosensitizer (e.g., 5-ALA), light, and molecular oxygen, leads to the generation of reactive oxygen species (ROS), which results in damage to cancer cells. ROS activate various regulated cell death pathways, including apoptosis, ferroptosis, and necroptosis. Furthermore, PDT induces immunogenic cell death by releasing DAMP (Damage-Associated Molecular Patterns) molecules (including calreticulin and HMGB1), which leads to the activation of dendritic cells and T lymphocytes. The therapy also affects the tumor microenvironment by modulating the expression of immune checkpoint molecules (e.g., PD-L1) and cytokines (*IL-6*, *TGF-β*). Additionally, changes in the expression of genes associated with oxidative stress (*SLC7A11*, *GPX4*), autophagy (*BECN1*, *ATG5*, *LC3B*), and stem cell markers (*SOX2*, *CD133*) influence the response to treatment and the development of resistance. These mechanisms justify the combination of PDT with immunotherapy and targeted therapies.

Analysis of gene expression in glioma cells treated with PDT highlights the importance of pathways associated with oxidative stress, autophagy, and metabolic adaptation. Genes such as *SLC7A11* and *GPX4* are responsible for maintaining redox balance and limiting lipid peroxidation, thereby influencing the cells’ susceptibility to ferroptosis. At the same time, autophagy genes, including *BECN1*, *ATG5*, and *LC3B*, participate in the cell’s stress response and can perform both cytoprotective and pro-apoptotic functions. Additionally, cancer stem cell markers such as *SOX2* and *CD133* are associated with treatment resistance and GBM recurrence (Table 1). These findings suggest that integrating PDT with molecularly targeted therapies may enhance treatment efficacy [21,22,23]. A schematic overview of the major molecular mechanisms involved in PDT-induced responses in glioblastoma is presented in Figure 1.

An extended list of genes potentially associated with glioblastoma biology and photodynamic therapy, together with their biological functions and supporting literature, is presented in Supplementary Table S1.

## 2. Materials and Methods

### 2.1. Cell Culture

The human glioblastoma cell line T98G (ATCC, Manassas, VA, USA) was used in this study. Cells were cultured under standard conditions at 37 °C in a humidified atmosphere containing 5% CO_2_. Cells were maintained in EMEM (Sigma-Aldrich, St. Louis, MO, USA) supplemented with 10% fetal bovine serum (Gibco, Thermo Fisher Scientific, Waltham, MA, USA) and 1% antibiotic mixture (penicillin–streptomycin–neomycin; Sigma-Aldrich, St. Louis, MO, USA). For all experiments, cells were seeded at in a 6-well plate (200,000 cells per well) and allowed to adhere prior to treatment.

### 2.2. Chemicals and Reagents

5-Aminolevulinic acid (Sigma-Aldrich, St. Louis, MO, USA) was used as a photosensitizer for photodynamic treatment. RNA isolation was performed using the RNeasy Mini Kit and RNase-Free DNase Set (Qiagen, Hilden, Germany). Reverse transcription was carried out using the High-Capacity cDNA Reverse Transcription Kit (Applied Biosystems, Foster City, CA, USA). Gene expression analysis was performed using TaqMan^®^ Gene Expression Assays and TaqMan^®^ Fast Advanced Master Mix (Applied Biosystems, Foster City, CA, USA). All procedures were performed according to the manufacturers’ instructions.

### 2.3. 5-ALA-Based Photodynamic Treatment (5-ALA-PDT)

For photodynamic treatment, T98G cells were incubated with 5-ALA at a final concentration of 0.015 M. A volume of 7 µL of the photosensitizer was added directly to the cell culture medium. Cells were incubated with 5-ALA for 3 h under standard culture conditions. Following incubation, cells were exposed to laser irradiation for 15 min to induce photodynamic activation. After treatment, cells were processed for downstream analyses.

### 2.4. 5-ALA Treatment Without Light Exposure

To assess the effects of 5-ALA alone, T98G cells were treated with 5-ALA without subsequent light exposure. Cells were incubated with 7 µL of 5-ALA at a concentration of 0.015 M under the same conditions as described above but were not subjected to laser irradiation. After the completion of the PDT protocol, RNA extraction was performed immediately without any recovery period, to capture acute transcriptional changes.

### 2.5. RNA Isolation

The T98G cell pellets were lysed using RLT buffer from the RNeasy Mini Kit. Total RNA was isolated according to the manufacturer’s instructions, including on-column DNase digestion using RNase-Free DNase Set to remove genomic DNA contamination. RNA concentration and purity were assessed spectrophotometrically using a NanoPhotometer Pearl (Implen, Munich, Germany). Purified RNA samples were stored at −80 °C until further analysis.

### 2.6. Reverse Transcription

Complementary DNA (cDNA) was synthesized from 10 ng of total RNA per sample using the High-Capacity cDNA Reverse Transcription Kit, according to the manufacturer’s protocol. Reverse transcription reactions were performed using a Mastercycler Personal Thermal Cycler (Eppendorf, Hamburg, Germany).

### 2.7. Quantitative Real-Time PCR Analysis

Gene expression analysis was performed using quantitative real-time PCR (RT-qPCR) with TaqMan^®^ Gene Expression Assays for the following genes: *GAPDH* (Hs99999905_m1), *ACSL4* (Hs00244871_m1), *GPX4* (Hs00157812_m1), *GCH1* (Hs00609198_m1), *SLC7A11* (Hs00921938_m1), *BAX* (Hs00180269_m1), *FAS* (Hs00236330_m1), *CASP3* (Hs00234387_m1), and *BCL2* (Hs00608023_m1).

Reactions were performed using TaqMan^®^ Fast Advanced Master Mix on a QuantStudio 5 Real-Time PCR System (Applied Biosystems, Foster City, CA, USA). Relative gene expression levels were calculated using the 2^−ΔΔCt^ method, with *GAPDH* as the endogenous control. Experiments were conducted in three independent biological replicates, and each reaction was performed in technical triplicates.

### 2.8. Treatment Protocol

Approximately 1 × 10^9^ cells/mL were seeded in culture plates and incubated with 5-ALA under dark conditions at 37 °C. 5-ALA was used as a precursor of protoporphyrin IX (PpIX), which acts as the active intracellular photosensitizer following metabolic conversion within the cells.

Cells were incubated with 5-ALA at the selected experimental concentration. Following incubation, excess unbound 5-ALA was removed by washing, and cells were further maintained in fresh medium prior to irradiation.

Photoactivation was performed using a laser/light source emitting light at a wavelength suitable for PpIX activation, within the red-light range. Irradiation was performed to induce photodynamic activation of intracellularly accumulated PpIX. Subsequently, downstream analyses were performed to assess the biological effects of 5-ALA-mediated photodynamic therapy.

5-ALA is widely used in neuro-oncology because of its preferential accumulation and conversion to PpIX in glioma cells. This enables both intraoperative fluorescence-guided visualization and photodynamic therapeutic approaches. Compared with many exogenous photosensitizers, 5-ALA is a prodrug that leads to endogenous PpIX accumulation, allowing selective targeting of tumor cells while relatively sparing normal tissues.

In this study, 5-ALA-based PDT was applied to evaluate the cellular response to photodynamic activation, with particular emphasis on oxidative stress, apoptosis, ferroptosis-related mechanisms, and gene expression changes.

### 2.9. Photodynamic Therapy

Cells were incubated with 5-ALA under dark conditions at 37 °C to allow intracellular accumulation of PpIX. Following incubation, the photosensitizer-containing medium was removed, and cells were washed with phosphate-buffered saline (PBS; EURx Molecular Biology Products, Gdańsk, Poland). The treated cultures were subsequently exposed to irradiation using a diode laser system (LED Diall IP54 0203H (Diall, London, UK)).

All irradiation procedures were conducted at room temperature (25 °C). The light source emitted radiation isotropically, with samples positioned at a distance of 20 cm from the emitter. The system operated at a nominal power of 400 W with an optical efficiency of 50%, resulting in an effective output power of 200 W. Under these conditions, the emitted light was distributed across the surface of a sphere with a radius of 20 cm, corresponding to an approximate total surface area of 5027 cm^2^:

A = 4π*r*^2^ = 4π(20)^2^ = 400π ≈ 5027 cm^2^

Given that the illuminated sample area was 1 cm^2^, the power density (irradiance) at the surface of the sample was determined as follows:

I = *P*/*A* = 200 W/5027 cm^2^ ≈ 0.0398 W/cm^2^

For an exposure duration of 15 min (900 s), the total light dose (fluence) at the sample surface was calculated based on the irradiance:

*H* = *I* × *t* = 0.0398 W/cm^2^ × 900 s ≈ 35.8 J/cm^2^

**Figure 2:**
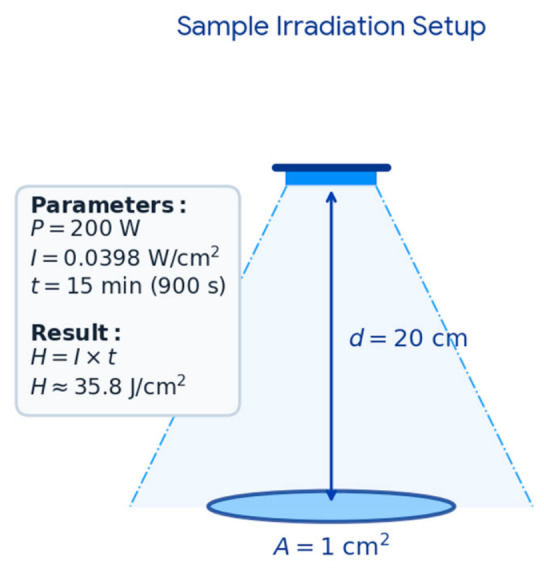
Schematic diagram of the irradiation setup for 5-ALA-mediated photodynamic therapy (5-ALA-PDT). Cells were incubated with 5-ALA under dark conditions, allowing intracellular accumulation of PpIX. After incubation, cells were rinsed with PBS prior to light exposure. Samples were irradiated using light of an appropriate wavelength for PpIX activation. The applied irradiation parameters enabled calculation of irradiance and total light dose delivered to the sample surface.

Under these conditions, 5-ALA-mediated photodynamic therapy resulted in activation of intracellular PpIX, leading to the generation of ROS. This induced oxidative stress and subsequent cellular damage, triggering molecular pathways associated with apoptosis, ferroptosis, and adaptive stress responses. A schematic overview of the experimental irradiation setup, including 5-ALA incubation, PBS washing, light exposure, and calculation of the irradiation parameters, is presented in Figure 2.

### 2.10. Cell Viability and Cell Concentration Count

Cell viability was assessed using the trypan blue exclusion assay (Sigma-Aldrich, Allentown, PA, USA), with cell counts performed manually using a hemocytometer (Hausser Scientific, Horsham, PA, USA).

For automated analysis, a 20 µL sample of the cell suspension was transferred into an Eppendorf tube and mixed with 380 µL of Muse^®^ Cell Count & Viability reagent (Luminex, Austin, TX, USA). The mixture was incubated at room temperature for 5 min. Subsequently, cell concentration (cells/mL) was determined using a Guava^®^ MUSE^®^ Cell Analyzer (Cytek Biosciences B.V., Amsterdam, The Netherlands).

### 2.11. Spectroscopic Measurements

Fluorescence emission measurements were performed to assess 5-ALA-induced intracellular accumulation of PpIX. Measurements were conducted using a fluorescence spectroscopic system according to the manufacturer’s guidelines.

PpIX fluorescence was monitored within its characteristic emission range after excitation with 635 nm wavelength. The measurements allowed evaluation of photosensitizer accumulation and photodynamic activation potential in treated cells.

Where applicable, singlet oxygen generation was assessed as an indicator of photodynamic activity. Singlet oxygen detection was performed using steady-state and/or time-resolved phosphorescence measurements, with emission monitored at approximately 1270 nm.

For phosphorescence spectra we used a fully automated fluorescence spectrometer FluoTime 300 EasyTau 2 software (version 2.3, PicoQuant, Berlin, Germany), equipped with options for phosphorescence measurements, including singlet oxygen emission in the NIR range. We used a picosecond-pulsed diode laser as the 5-Ala excitation source. The emission was collected at an angle of 90° using an NIR-sensitive detector with a monochromator. The EasyTau 2 software automated the setup, i.e., wavelength selection, slit adjustment (10 μm–4 mm), spectrum acquisition, and kinetics. The data was analyzed in FluoFit (version 4.2, PicoQuant, Berlin, Germany) with exponential fitting and quality assessment. To optimize sensitivity and minimize noise, the PMT detector was thermoelectrically cooled to reduce dark counts. We used long-pass filters (scattered light rejection 1:105–1:1010) and overload protection. The monochromator was set to 1270 nm with a resolution of ≤0.1 nm and with NIR-optimized gratings. The sample was placed in a 1 cm cuvette in a multifunctional chamber at a temperature of 15 °C. The entire setup was controlled by software—there were no manual adjustments of the optics during measurement.

Instrument operation, including wavelength selection, spectral acquisition, and kinetic measurements, was software-controlled. Data processing was performed using appropriate fitting and analysis software. Background signal reduction and detector sensitivity optimization were applied to improve measurement accuracy.

**Figure 3:**
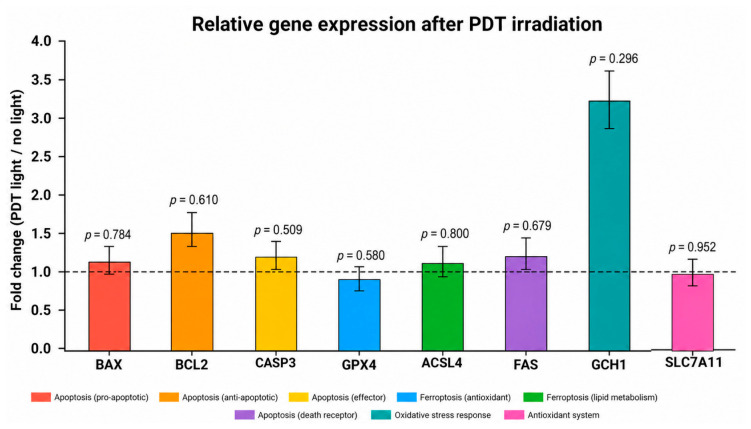
Fold change in gene expression after PDT irradiation relative to no-light control conditions. The dashed line indicates no change (fold change = 1.0). *p*-values are shown above each bar and should be interpreted as exploratory due to the limited sample size (*n* = 3 per group).

**Figure 4:**
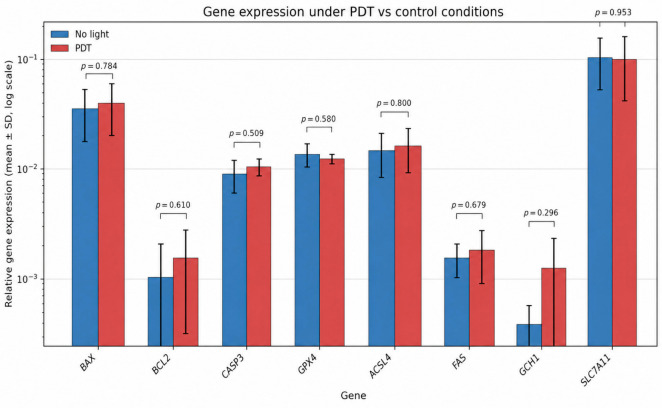
Relative gene expression levels in PDT/light-treated and no-light control samples. Bars represent mean ± SD. A logarithmic y-axis was used to improve visualization of genes with low baseline expression.

Samples were placed in suitable cuvettes or culture dishes depending on the measurement protocol. All measurements were performed under controlled experimental conditions to ensure reproducibility. The effects of photodynamic treatment on the expression of apoptosis-, ferroptosis-, and oxidative stress-related genes were evaluated separately by gene expression analysis. The fold changes relative to the no-light control are summarized in Figure 3. Relative gene expression levels in PDT-treated and no-light control samples are presented in Figure 4.

### 2.12. Statistical Analysis

Data are reported as mean ± standard deviation. Differences levels in *T98* cells and healthy cells were calculated by Welch *t*-test at *p* < 0.05, using Statistica version 13.3 (TIBCO Software Inc., Palo Alto, CA, USA). The data were presented through Microsoft Excel 365 (Microsoft Corporation, Redmond, WA, USA).

Gene expression values were compared between PDT/light-treated samples and no-light controls. Mean values, standard deviations (SD), fold changes, log2 fold changes, and two-tailed Welch *t*-test *p*-values were calculated for each gene using three biological/technical replicates per condition. Because *n* = 3 per group, *p*-values should be interpreted cautiously and treated as exploratory unless supported by additional replicates.

**Figure 5:**
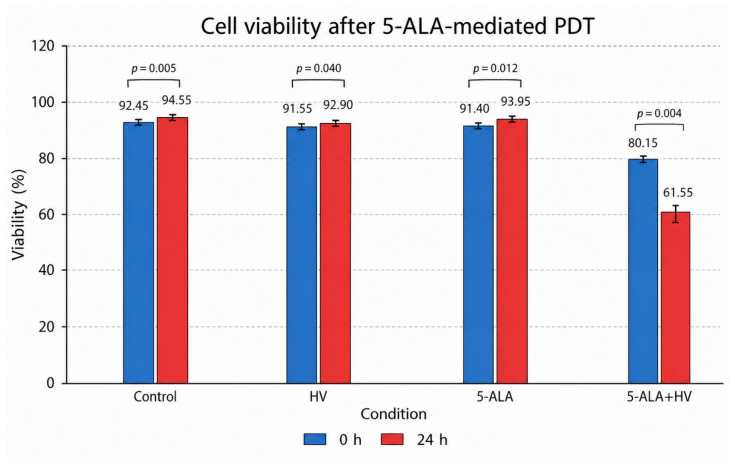
Cell viability in Control, HV, 5-ALA, and 5-ALA+HV groups at 24 h post-treatment. Values represent mean ± SD (*n* = 3). Statistical significance was assessed using Welch’s two-sample *t*-test. The indicated *p*-values correspond to pairwise comparisons between the 5-ALA+HV (PDT) group and the respective control groups. A substantial decrease in viability was observed only in the 5-ALA+HV group, confirming the cytotoxic effect of photodynamic therapy.

Cell viability data were obtained from three independent experiments (*n* = 3) and are presented as mean ± standard deviation (SD). Statistical comparisons between experimental groups (Control, HV, 5-ALA, and 5-ALA + HV) at each time point were performed using Welch’s two-sample *t*-test. The *p*-values shown in Figure 4 correspond to pairwise comparisons of the 5-ALA+HV (PDT) group versus the Control, HV, and 5-ALA groups. Statistical significance was defined as *p* < 0.05 (Figure 5).

## 3. Results

### 3.1. Gene Expression Profiling and Fold-Change Analysis Following PDT

Gene expression analysis revealed moderate but consistent changes following PDT compared to the control group that did not receive irradiation. The fold changes in expression and a statistical summary of all analyzed genes are presented in Table 2.

### 3.2. Fold-Change Analysis

Overall, PDT induced a trend toward upregulation of several genes associated with apoptosis and stress response.

Pro-apoptotic markers, including *BAX* (fold change: 1.13), *CASP3* (1.17), and *FAS* (1.18), showed a modest increase following irradiation. These findings suggest activation of apoptotic pathways in response to PDT-induced oxidative stress.

The anti-apoptotic gene *BCL2* demonstrated a higher fold change (1.50), indicating that, alongside pro-death signaling, cells simultaneously activate survival mechanisms.

Among ferroptosis-related genes, *ACSL4* was slightly upregulated (1.10), while *GPX4* expression decreased (0.91), suggesting a shift toward increased susceptibility to lipid peroxidation. In contrast, *SLC7A11* expression remained relatively unchanged (0.97), indicating preservation of cellular antioxidant defense systems.

The most pronounced change was observed for *GCH1* (fold change: 3.24; log2FC: 1.70), reflecting a strong activation of oxidative stress response pathways.

PDT induced observable trends in gene expression profile associated with apoptosis, ferroptosis, the oxidative stress response, and treatment adaptation compared to control conditions (without irradiation).

Among the genes associated with apoptosis, an increase in *BAX* expression was observed from 0.0355 ± 0.0178 in the control group to 0.0401 ± 0.0200 after PDT, corresponding to a 1.13-fold increase. Similarly, *CASP3* showed a moderate increase from 0.00895 ± 0.00296 to 0.0104 ± 0.00188 (1.17-fold). *FAS* expression also increased from 0.00154 ± 0.00052 to 0.00182 ± 0.00091 (1.18-fold). These results suggest moderate activation of proapoptotic pathways in response to photodynamic therapy.

At the same time, the anti-apoptotic *BCL2* gene was also upregulated (from 0.00103 ± 0.00104 to 0.00154 ± 0.00122; 1.50-fold), indicating that survival mechanisms may be activated in parallel with the processes leading to cell death.

Genes associated with ferroptosis exhibited varied expression patterns. *ACSL4*, which is involved in lipid remodeling and susceptibility to lipid peroxidation, showed an increase in expression from 0.0147 ± 0.00635 to 0.0162 ± 0.00706 (1.10-fold). In contrast, *GPX4* decreased from 0.0136 ± 0.00328 to 0.0123 ± 0.00125 (0.91-fold). Since GPX4 is the primary inhibitor of lipid peroxide accumulation, its decrease may indicate increased cellular susceptibility to ferroptosis.

*SLC7A11* expression remained relatively stable (fold change = 0.97), suggesting that antioxidant mechanisms related to cystine transport and glutathione synthesis were not significantly inhibited under the tested conditions.

The most pronounced change was observed for *GCH1*, whose expression increased from 0.000386 ± 0.000190 to 0.00125 ± 0.00107 (3.24-fold). Such a significant increase indicates the activation of adaptive mechanisms related to oxidative stress and the regulation of redox balance.

Despite the observed changes, Welch’s *t*-test did not reveal statistical significance for any of the analyzed genes (*p* > 0.05), which is most likely due to the small number of replicates and the variability of the results. Therefore, the obtained data should be treated as preliminary trends requiring further validation.

### 3.3. Cell Viability Analysis (MUSE^®^ Cell Analyzer)

Cell viability was assessed using the MUSE^®^ Cell Analyzer in three independent replicates (*n* = 3) at two time points: immediately after treatment (0 h) and after 24 h. The results are summarized in Table 3.

At 0 h, cell viability remained high and comparable in the control, HV, and 5-ALA groups (≈91–92%), indicating that neither 5-ALA alone nor light exposure alone caused immediate cytotoxicity. In contrast, the 5-ALA + HV (PDT) group showed a noticeable decrease in viability (80.15%), suggesting an early phototoxic effect.

After 24 h, viability in the control, HV, and 5-ALA groups remained stable or increased slightly (≈93–95%), confirming the absence of delayed cytotoxicity under these conditions. However, a significant decrease in viability (61.55%) was observed in the PDT group, indicating a strong, time-dependent cytotoxic effect of photodynamic therapy using 5-ALA.

These results show that PDT induces significant cell death, which becomes more pronounced over time, consistent with mechanisms involving oxidative stress and delayed activation of cell death pathways.

**Figure 6:**
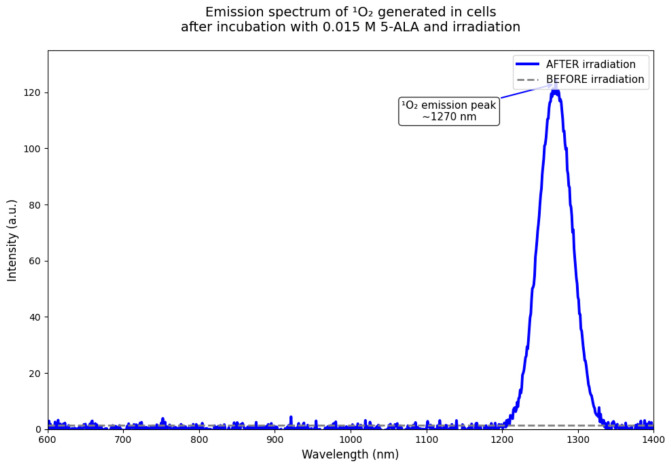
Emission spectrum of singlet oxygen generated in T98G cells after 5-ALA PDT.

The spectroscopic findings (Figure 6) demonstrate that a 0.015 M 5-ALA solution serves as an exceptionally efficient photosensitizer in T98G glioblastoma cells. The symmetrical 1270 nm emission peak—free from overlapping background bands—confirms the high specificity of singlet-oxygen detection in vitro. Consequently, this concentration enables robust, targeted generation of reactive oxygen species (ROS), validating 5-ALA as a potent candidate for near-infrared photodynamic therapy (PDT).

## 4. Discussion

In a study conducted by Mastrangelopoulou et al., the key regulator of ferroptosis—glutathione peroxidase 4 (*GPX4*)—exhibited the highest expression in U87 cells compared to *T47D*, *MCF7*, T98G, and *MDA-MB-231* cells. Only in the case of T98G cells was the decrease in the expression of this gene between the groups of cells treated with 5-ALA alone and 5-ALA-PDT statistically significant [8]. Karmakar et al., conducting research on U87MG cells, analyzed the mechanisms involved in PDT-induced apoptosis. The Bax:Bcl-2 ratio indicates the degree of a cell’s involvement in apoptosis. The researchers observed a 235% and 267% increase in this ratio compared to the control group at 0.5 and 1 mM 5-ALA-PDT, respectively. Additionally, an increase in the activation of caspase-3 and caspase-9—factors involved in caspase-dependent apoptosis—was also observed [26].

Inoue et al. also investigated caspase activity following PDT. After 5-ALA-PDT was applied to U251MG cells, a significant increase in caspase-3 and caspase-9 activity was observed, with no effect on caspase-8 activity, suggesting predominant involvement of the mitochondrial apoptotic pathway [24]. In the present study, modest increases in the expression of apoptosis-related genes were observed following PDT treatment. Although these expression patterns may be consistent with activation of multiple cell death–related pathways, none of the analyzed gene expression changes reached statistical significance. Therefore, the observed trends should be considered exploratory and interpreted with caution until confirmed in larger studies and functional assays [41,42].

A moderate increase in the expression of *BAX*, *CASP3*, and *FAS* may be consistent with activation of apoptotic pathways; however, these observations should be interpreted cautiously because none of the expression changes reached statistical significance. Mechanistically, this can be explained by the action of reactive oxygen species (ROS) generated during PDT, which lead to mitochondrial damage, the release of cytochrome c, and the activation of the caspase cascade [41,43]. The increase in *CASP3* expression may suggest activation of apoptotic signaling, while the increase in *FAS* expression may indicate potential involvement of the extrinsic apoptotic pathway [41].

At the same time, increased *BCL2* expression may reflect activation of cellular defense mechanisms. BCL2 may limit the mitochondrial apoptotic pathway and influence the cellular response to PDT [28].

The expression profile of ferroptosis-related genes may be consistent with an altered cellular response to oxidative stress following PDT. In the present study, *ACSL4* expression showed a modest increase (fold change = 1.12), whereas *GPX4* expression was moderately reduced (fold change = 0.87). Although neither change reached statistical significance, this expression pattern may be compatible with increased susceptibility to lipid peroxidation and ferroptotic cell death, as *ACSL4* has been associated with the incorporation of polyunsaturated fatty acids into membrane phospholipids, while GPX4 plays a central role in protecting cells against lipid peroxidation [16,44]. In contrast, *SLC7A11* expression remained largely unchanged (fold change = 0.99), suggesting that cystine transport and glutathione-related antioxidant defenses were not substantially affected under the experimental conditions. Taken together, these observations may indicate a tendency toward ferroptosis-related signaling; however, due to the lack of statistical significance and the absence of functional validation, these findings should be interpreted with caution [16,45].

An interesting observation was the increase in *GCH1* expression, which is associated with the regulation of redox balance and protection against lipid peroxidation [36,40,46,47].

Taken together, the results suggest that PDT may modulate both cytotoxic and adaptive response pathways, which may limit the efficacy of the therapy [41,42].

The results of the cell viability assay further corroborate the findings regarding gene expression and provide functional confirmation of the cytotoxicity induced by PDT. While the gene expression analysis revealed only moderate and statistically insignificant changes, the viability assay demonstrated a clear biological effect of the treatment [2,48].

The absence of cytotoxicity in the group receiving 5-ALA alone confirms that 5-ALA itself is not toxic in the absence of light activation, which is consistent with its role as a prodrug [2,49]. Similarly, irradiation (HV) alone did not significantly affect cell viability. In contrast, the combination of 5-ALA and light irradiation caused a significant reduction in viability, particularly after 24 h, indicating that photodynamic activation is necessary to induce cell death [2,50].

The delayed decline in viability suggests that PDT-induced cytotoxicity is not immediate but develops over time, likely due to the accumulation of ROS, mitochondrial damage, and the subsequent activation of apoptotic and ferroptotic pathways [2,48,51]. This is consistent with the observed upregulation of proapoptotic genes, such as *BAX* and *CASP3*, as well as the modulation of genes associated with ferroptosis, including *GPX4* and *ACSL4* [16,51].

Interestingly, despite a marked reduction in cell viability following PDT, particularly at 24 h (61.55% viability in the 5-ALA+HV group compared with >92% in the control groups), the observed changes in gene expression remained relatively modest and did not reach statistical significance. For example, pro-apoptotic genes such as *BAX*, *CASP3*, and *FAS* showed only slight increases in expression (fold changes ranging from 1.13 to 1.18), whereas the anti-apoptotic gene *BCL2* also exhibited increased expression (fold change = 1.50). Similarly, ferroptosis-related genes demonstrated only moderate alterations, including a slight increase in *ACSL4* expression and a modest decrease in *GPX4*. These findings suggest that the substantial loss of cell viability may not be fully explained by transcriptional changes alone. Instead, post-transcriptional mechanisms, protein-level regulation, rapid oxidative damage to cellular structures, or other molecular events occurring downstream of gene transcription may contribute significantly to PDT-induced cytotoxicity [48,52]. Nevertheless, these interpretations remain speculative and require confirmation through functional and protein-level analyses.

Overall, a combined analysis of gene expression and cell viability suggests that PDT using 5-ALA induces a complex, time-dependent cytotoxic response involving multiple overlapping mechanisms, including apoptosis, ferroptosis, and oxidative stress-induced damage [2,51,52].

This study has several limitations that should be acknowledged. First, the experiments were performed using a single glioblastoma cell line (T98G), which may not fully reflect the biological heterogeneity of glioblastoma. Second, the analysis was limited to mRNA expression and did not include protein-level validation or functional assays assessing apoptosis, ferroptosis, oxidative stress, or related signaling pathways. Third, only a single PDT condition was evaluated, and no dose–response or time-course analyses were performed to assess dynamic changes in gene expression following treatment. Finally, the findings were not validated in animal models or other in vivo systems. Therefore, the present results should be considered preliminary and hypothesis-generating, requiring confirmation in additional glioblastoma models, functional studies, and in vivo investigations.

## 5. Conclusions

The present study demonstrates that 5-ALA-mediated PDT induces both cytotoxic and adaptive responses in glioblastoma cells. Although gene expression changes associated with apoptosis, ferroptosis, and oxidative stress did not reach statistical significance, a marked time-dependent reduction in cell viability confirmed the biological effect of PDT.

These findings suggest that PDT may simultaneously activate cell death and survival-related pathways. However, due to the limited number of replicates and the lack of functional validation, the results should be considered preliminary. Further studies are needed to confirm the molecular mechanisms underlying the response to PDT and to evaluate its therapeutic potential.

## Figures and Tables

**Table 1:** Genes analyzed in this study and their biological relevance in 5-ALA-mediated photodynamic therapy (PDT) of glioblastoma.

Gene	Full Name	Biological Process	Relevance to PDT Response	Suggested References
*BAX*	*BCL2*-associated X protein	Apoptosis	Promotes mitochondrial apoptosis and PDT-induced cell death	[24,25,26,27]
*BCL2*	*BCL2* apoptosis regulator	Apoptosis/Cell survival	Inhibits apoptosis and supports cell survival after oxidative stress	[24,25,26,28]
*CASP3*	Caspase 3	Apoptosis	Key executioner caspase responsible for apoptotic cell death	[22,23,24,29,30,31]
*FAS*	*FAS* cell surface death receptor	Extrinsic apoptosis	Activates death receptor-mediated apoptotic signaling	[27,32]
*ACSL4*	Acyl-CoA synthetase long-chain family member 4	Ferroptosis	Increases susceptibility to lipid peroxidation and ferroptotic cell death	[16,33]
*GPX4*	Glutathione peroxidase 4	Ferroptosis/Redox regulation	Protects cells from lipid peroxidation and suppresses ferroptosis	[34,35,36]
*SLC7A11*	Solute carrier family 7 member 11	Redox homeostasis	Regulates cystine uptake and glutathione synthesis, contributing to antioxidant defense	[37,38,39]
*GCH1*	GTP cyclohydrolase 1	Oxidative stress response	Regulates cellular adaptation to oxidative stress and ferroptosis resistance	[40]

**Table 2:** Fold-change and statistical summary. Fold change was calculated as PDT/light mean divided by no-light mean. *p*-values were calculated using Welch’s two-sample *t*-test.

Gene	PDT Mean ± SD	No Light Mean ±SD	Fold Change	log2FC	*p*-Value	Direction
*BAX*	0.04007 ± 0.02	0.03554 ± 0.0178	1.1274	0.1729	0.7844	Up
*BCL2*	0.001544 ± 0.00122	0.001031 ± 0.00104	1.4974	0.5824	0.6103	Up
*CASP3*	0.01044 ± 0.00188	0.008947 ± 0.00296	1.1667	0.2225	0.5091	Up
*GPX4*	0.01232 ± 0.00125	0.0136 ± 0.00328	0.9060	−0.1424	0.5803	Down
*ACSL4*	0.01616 ± 0.00706	0.01467 ± 0.00635	1.1011	0.1389	0.8003	Up
*FAS*	0.001818 ± 0.00091	0.001544 ± 0.000516	1.1778	0.2361	0.6787	Up
*GCH1*	0.001253 ± 0.00107	0.0003863 ± 0.00019	3.2425	1.6971	0.2957	Up
*SLC7A11*	0.1016 ± 0.0598	0.1045 ± 0.0521	0.9722	−0.0407	0.9525	Stable

**Table 3:** Cell viability (%) following 5-ALA and 5-ALA-PDT treatment (mean values, *n* = 3).

Viability (*n* = 3)	Control	HV	5-ALA	5-ALA+HV
0 h	92.45 ± 0.00	91.55 ± 0.32	91.40 ± 0.09	80.15 ± 0.18
24 h	94.55 ± 0.27	92.90 ± 0.60	93.95 ± 0.53	61.55 ± 2.11

## Data Availability

The original contributions presented in this study are included in the article and supplementary material. Further inquiries can be directed to the corresponding author.

## Supplementary Materials

The following supporting information can be downloaded at: https://www.mdpi.com/article/10.3390/cimb48070689/s1, Table S1. Comprehensive overview of genes potentially involved in glioblastoma biology and photodynamic therapy response [53,54,55,56,57,58,59,60,61,62,63,64,65,66,67,68,69,70,71,72,73,74,75,76,77,78,79].
