# Supplementary material for: 5-ALA Photodynamic Therapy Induces Competing Death and Survival Pathways in Glioblastoma Cells
Source: Curr Issues Mol Biol. 2026 Jul 3;48(7):689. doi: 10.3390/cimb48070689 (PMC13407687; doi:10.3390/cimb48070689)
Supplement: Supplementary file 1 [file cimb-48-00689-s001.zip › cimb-4382300-supplementary.pdf]

Supplementary Materials:

Table S1. Comprehensive overview of genes potentially involved in glioblastoma biology and photodynamic therapy response.

No.	Symbol of gene	Full name of gene	Cytogenetic location	Encoded product	Function	Suggested references
1.	FECH	Ferrochelatase	18q21.31	FECH - Ferrochelatase	Heme biosynthesis enzyme, catalyzes the incorporation of iron ions into Pp IX, regulating PpIX accumulation and influencing cell sensitivity to 5-ALA-PDT.	[2, 8, 12, 61]
2.	HMOX1	Heme oxygenase-1	22q12.3	HO-1 - heme oxygenase-1	An enzyme that breaks down heme into biliverdin, iron and carbon monoxide, protecting cells from oxidative stress and playing a role in the regulation of cytoprotective processes.	[2, 8, 12]
3.	SLC15A1	Solute carrier family 15 member 1	13q32.3 [OO]	PepT1 - H^+-dependent peptide transporter 1	Proton-dependent peptide transporter responsible for the uptake of di- and tripeptides. Although its direct role in glioblastoma and 5-ALA-PDT remains unclear, peptide transporters may influence the cellular uptake of small molecules and could represent a potential exploratory target in drug delivery strategies.	[2, 62]
4.	ABCG2	ATP-binding cassette, sub-family G, member 2	4q22.1	ABCG2 - ATP-binding cassette sub-family G member 2	A membrane transporter involved in the transport of biological molecules across cell membranes, actively pumping various substances out of the cell, including protoporphyrin IX, and regulating the intracellular accumulation of porphyrin derivatives.	[63]
5.	ABCB1	ATP-binding cassette, subfamily B, member 1	7q21.12	ABCB1 - ATP-binding cassette sub-family B member 1	A membrane transporter that actively exports xenobiotic compounds from cells, playing a key role in detoxification and protecting tissues against their accumulation.	[2, 12]
6.	HIF-1α	Hypoxia-inducible factor 1, alpha subunit	14q23.2	HIF-1α - hypoxia inducible factor 1 subunit alpha	Transcription factor, regulates the expression of genes in response to cellular hypoxia, including those related to angiogenesis, metabolism and adaptation to oxygen stress.	[62, 64]
7.	VEGFA	Vascular endothelial growth factor A	6p21.1	VEGFA - vascular endothelial growth factor A	A growth factor that stimulates the formation of new blood vessels, increases cell proliferation and motility, and regulates vascular permeability.	[62, 64]

8.	SLC2A1	Solute carrier family 2, member 1	1p34.2	GLUT1 - glucose transporter 1	Glucose transporter enabling the absorption of glucose from the blood into cells, including across the blood-brain barrier, providing the basic source of energy for cellular metabolism.	[62, 64]
9.	CA IX	Carbonic anhydrase IX	9p13.3	CA IX - carbonic anhydrase IX	An enzyme that catalyzes the conversion of carbon dioxide and water into bicarbonate ions and protons, acidifying the tumor microenvironment, promoting the migration and invasion of cancer cells.	[62]
10.	LDHA	Lactate dehydrogenase A	11p15.1	LDHA - Lactate dehydrogenase A	An enzyme that catalyzes the reversible conversion of pyruvate to lactate, enabling glycolysis and energy production to be maintained under conditions of limited oxygen availability.	[62]
11.	BNIP3	BCL2 and adenovirus E1B 19 kDa-interacting protein 3	10q26.3	BNIP3 - BCL2 and adenovirus E1B 19 kDa-interacting protein 3	A protein involved in the regulation of cell death, autophagy and mitophagy, especially under conditions of cellular stress and hypoxia.	[62, 65]
12.	MMP2	matrix metallopeptidase 2	16q12.2	MMP2 - Matrix metallopeptidase 2	An enzyme involved in the degradation of the extracellular matrix, contributing to tumor infiltration and invasion.	[62]
13.	NFE2L2	Nuclear factor, erythroid 2 like 2	2q31.2	NRF2 - NF-E2-related factor 2	A protein regulating antioxidant enzymes, responsible for the response to oxidative stress and modulating inflammatory responses.	[12, 37]
14.	GPX4	Glutathione peroxidase 4	19p13.3	GPX4 - Glutathione peroxidase 4	A selenium-dependent enzyme that protects cells against oxidative stress and lipid peroxidation and inhibits ferroptosis, which helps maintain the integrity of cell membranes and prevent iron-dependent cell death.	[37, 38]
15.	SOD2	Superoxide dismutase-2	6q25.3	SOD2 - Superoxide dismutase-2	An enzyme that protects cells from oxidative stress by detoxifying superoxide radicals.	[2]
16.	SOX2	SRY-box transcription factor 2	3q26.33	SOX2 - SRY-box transcription factor 2	A transcription factor that controls the processes of differentiation and self-renewal using transcription programs.	[21, 66]
17.	PROM1 (CD133)	Prominin 1	4p15.32	PROM1 - Prominin 1	A protein that acts as an organizer of microvilli and cell protrusions, regulating the structure and dynamics of the cell membrane and influencing cellular processes such as proliferation,	[21, 66]

				differentiation, migration, autophagy and carcinogenesis.	
18.	BAX	BCL2-associated X protein, apoptosis regulator	19q13.33	BAX - BCL2-associated X protein	Pro-apoptotic protein, promotes cell death by initiating the release of pro-apoptotic factors from mitochondria.	[24 – 26]
19.	BCL2	BCL2, apoptosis regulator	18q21.33	BCL2 - BCL2	Anti-apoptotic protein, inhibits cell death by supporting survival signals and mitochondrial stability.	[24 – 26]
20.	CASP3	Caspase 3	4q35.1	CASP3 - Caspase-3	The executive protease of apoptosis triggers the process of protein degradation and leads to the completion of cell death.	[26, 29 – 31]
21.	CASP8	Caspase 8	2q33.1	CASP8 - Caspase-8	A cell death-initiating caspase that determines the course of apoptosis and necroptosis through interaction with RIPK1 and other signaling proteins.	[30,67, 68]
22.	RIPK1	Receptor-interacting ser-ine/threonine kinase 1	6p25.2	RIPK1 - Re-cep-tor-interacting ser-ine/threonine-protein kinase 1	Kinase regulating inflammation, cell proliferation and cell death, including apoptosis and necrosis, by interacting with other signaling proteins.	[31, 50, 69]
23.	RIPK3	Receptor-interacting serine/threonine kinase 3	4q12	RIPK3 - Receptor-interacting serine/threonine-protein kinase 3	A kinase that regulates programmed cell necrosis, initiating a caspase-independent cell death process.	[31, 67, 69]
24.	MLKL	Mixed line-age kinase domain-like protein	16q23.1	MLKL - Mixed lineage kinase domain-like protein	The protein, which after phosphorylation by RIPK3 undergoes conformational activation, oligomerizes and translocates to the cell membrane, triggering proinflammatory cell death in the process of necroptosis.	[67, 68, 70]
25.	BECN1	Beclin-1	17q21.31	BECN1 - Beclin-1	A key regulator of autophagy, it initiates the formation of autophagosomes and influences the balance between autophagy, apoptosis and the immune response.	[71, 72]
26.	LC3	Microtubule-associated protein 1, light chain 3	20q11.22	LC3 - Microtubule-associated protein 1, light chain 3	A key protein in LAP-associated phagocytosis, it promotes phagosome maturation, fusion with lysosomes, and degradation of engulfed material in response to pathogens.	[72]
27.	ATG5	Autophagy-related 5	6q21	ATG5 - Autophagy-related 5	The main component of autophagy, through the formation of the ATG12–ATG5–ATG16L1 complex, regulates the formation of autophagosomes.	[72]
28.	PD-L1	Programmed cell death 1 ligand 1	2q37.3	PDL1 - Programmed cell death 1 ligand 1	PD-1 receptor ligand, its expression in tissues is associated with the inhibition of T lymphocyte activity and modulation of their infiltration in the immune microenvironment.	[73 – 75]

29.	PD-L2	Programmed cell death 1 ligand 2	9p24.1	PDL2 - Programmed cell death 1 ligand 2	PD-1 ligand participates in the regulation of T lymphocyte responses, influencing the degree of their activation and presence in tissues.	[75, 76]
30.	TGFB1	Transforming growth factor beta 1	19q13.2	TGFB1 - Transforming growth factor beta 1	A signaling protein that regulates cell growth, differentiation, and apoptosis. It plays a crucial role in body development, tissue repair, and disease processes.	[75, 77]
31.	IL6	Interleukin 6	7p15.3	IL6 - Interleukin 6	A cytokine produced by immune cells in response to infection or tissue damage and coordinating the immune, hematological and acute phase responses, contributing to host defense and tissue regeneration.	[75, 78]
32.	IDO1	Indoleamine 2,3-dioxygenase-1	8p11.21	IDO1 - Indoleamine 2,3-dioxygenase-1	An enzyme that catalyzes the degradation of L-tryptophan to N-formyl-kynurenine.	[79, 80]
33.	CCL2	C-C motif chemokine ligand 2	17q12	CCL2 - C–C motif chemokine ligand 2	A chemokine responsible for the recruitment of monocytes, memory T cells and dendritic cells to sites of tissue damage and inflammation.	[81, 82]
34.	CXCL12	C-X-C motif chemokine ligand 12	10q11.21	CXCL12 - C-X-C motif chemokine ligand 12	A chemokine involved in the regulation of cell migration and signaling, important for developmental processes and tissue function.	[83 – 85]
35.	CALR	Calreticulin	19p13.13	CALR - Calreticulin	A multifunctional protein that binds and stores calcium in the endoplasmic reticulum and regulates gene transcription in the cell nucleus by interacting with hormone receptors.	[86, 87]
36.	HMGB1	High mobility group box 1	13q12.3	HMGB1 - High mobility group box 1	A non-histone nuclear protein that regulates chromatin structure and transcription. When released from cells, it acts as a mediator of the inflammatory response.	[86, 87]
37.	IFNB1	Interferon beta 1	9p21.3	IFNB1 - Interferon beta 1	A cytokine involved in the immune response, regulating antiviral and anti-inflammatory responses.	[75, 86]
38.	ACSL4	Acyl-CoA synthetase long chain family member 4	Xq23	LACS - Long chain acyl-CoA synthetase	An enzyme that catalyzes the activation of arachidonic acid and adrenoic acid by binding coenzyme A, increasing the susceptibility of cells to ferroptosis.	[16, 35]
39.	FAS	Fas cell surface death receptor	10q23.31	FAS - Fas cell surface death receptor	A receptor that, when activated by the Fas ligand, initiates the extrinsic apoptosis pathway, playing a key role in regulating the elimination of cancer cells by the immune system.	[32, 33]
40.	GSX1	GS homeobox 1	13q12.2	GSX1 - GS homeobox 1	A transcription factor that regulates the maturation of neural progenitors and the specification of neuronal cells	exploratory / lack of robust

					during the development of the central nervous system.	direct data on GBM/PDT
41.	SLC7A11	Solute carrier family 7 member 11	4q28.3	SLC7A11 - Solute carrier family 7 member 11	A membrane cystine transporter that regulates the redox balance in the cell and influences its survival or death by controlling cystine import and the level of disulfide stress.	[40, 42]
